# In Situ Direct Monitoring of the Morphological Transformation of Single Au Nanostars Induced by Iodide through Dual-Laser Dark-Field Microscopy: Unexpected Mechanism and Sensing Applications

**DOI:** 10.3390/nano12152555

**Published:** 2022-07-25

**Authors:** Weizhen Xu, Hongmei Luo, Min Ouyang, Tiantian Long, Qinlu Lin

**Affiliations:** National Engineering Laboratory for Rice and By-Products Further Processing, College of Food Science and Engineering, Central South University of Forestry & Technology, Changsha 410004, China; 20180100023@csuft.edu.cn (W.X.); 20201200556@csuft.edu.cn (H.L.); 20201200547@csuft.edu.cn (M.O.); 20191200447@csuft.edu.cn (T.L.)

**Keywords:** single nanoparticle imaging, Au nanostars, laser, dark-field microscopy, Green/Red channel intensities (G/R ratios)

## Abstract

Single nanoparticle imaging is a significant technique to help reveal the reaction mechanism and provides insight into the nanoparticle transformation. Here, we monitor the in situ morphological transformation of Au nanostars (GNSs) induced by iodide (I^−^) in real time using dark-field microscopy (DFM) with 638 nm red (R) and 534 nm green (G) laser coillumination. The two lasers are selected because the longitudinal localized surface plasmon resonance of GNSs is located at 638 nm and that for GNSs after transformation is at 534 nm. Interestingly, I^−^ can interact with GNSs directly without the engagement of other reagents, and upon increasing I^−^ concentrations, GNSs undergo color changes from red to orange, yellow, and green under DFM. Accordingly, green/red channel intensities (G/R ratios) are extracted by obtaining red and green channel intensities of single nanoparticles to weigh the morphological changes and quantify I^−^. A single nanoparticle sensor is constructed for I^−^ detection with a detection limit of 6.9 nM. Finally, a novel mechanism is proposed to elucidate this shape transformation. I^−^ absorbed onto the surface of GNSs binds with Au atoms to form AuI^−^, lowering the energy of its bond with other Au atoms, which facilitates the diffusion of this atom across the nanoparticle surface to low-energy sites at the concaves, thus deforming to spherical Au nanoparticles.

## 1. Introduction

Real-time imaging of single nanoparticles during their reactions and motions is vital to understanding their chemical [1,2,3,4], physical [5,6], and biological properties [7,8]. Although techniques such as atomic force microscopy (AFM) [9,10,11], liquid cell transmission electron microscopy (TEM) [12,13,14], and scanning electron microscopy (SEM) [15,16] are useful for monitoring single nanoparticles, they are expensive, exhibit low temporal resolution, and typically cannot be conducted under biophysical conditions. Thus, inadequate detailed kinetic information, inaccurate physical and chemical properties, and deactivation of biological function are problematic. Many techniques enabling the monitoring of real-time and in situ dynamic behaviors of single particles based on their optical properties have become important tools for understanding the physical and chemical properties and bifunctionality of nanoparticles [17,18,19]. Dark-field microscopy (DFM) is innately suitable for monitoring single plasmonic nanoparticles with a high signal-to-noise ratio and high spatial and temporal resolution. It is extensively applied in imaging [20,21], sensing [22,23,24], and drug and gene carriers [25]. It demonstrates promising prospects for understanding the details and mechanisms of certain chemical phenomena [26,27,28]. For example, DFM can monitor the transverse etching of Au nanorods (GNRs) using HAuCl_4_ at an arbitrary time in the presence of cysteine during the formation of Au nanodumbbells [29]. DFM was employed to examine the in situ formation of individual Ag@Hg nanoalloys where Hg atoms were rapidly adsorbed onto Ag nanoparticles and diffused into Ag cores to form Ag@Hg alloys [30]. When linear sweep potentials were applied, dynamic structural and morphological changes during the formation of Hg amalgamation on individual GNRs were observed via DFM, thus confirming the correlation between spectral changes and the formation of an amalgam [31]. Dynamic structural and morphological changes in Ag nanoparticles with the introduction of Au^3+^ via a galvanic exchange process were monitored in real time using DFM, indicating the formation of Ag–Au alloys as the intermediate state [32,33]. During this reaction, the color of the nanoparticle solution changed from blue to yellow and to dim red.

Iodine is one of the essential trace elements in the human body, which plays an important role in human growth and metabolism [34]. Iodine deficiency in the body would cause goiters and abnormal growth and development of the nervous system in children, resulting in different degrees of cretinism in adults, and abundance would induce hypothyroidism and hyperthyroidism health problems [35,36,37,38]. On the other hand, the risks of iodine poisoning continually persist. High intakes can lead to the occurrence of thyroid papillary carcinoma and acute poisoning symptoms [39,40]. Therefore, it is of great significance to realize the rapid, sensitive, and selective monitoring of iodide in food.

In addition, iodide (I^−^) has unique interactions with Au. It can be spontaneously chemisorbed on Au surfaces primarily because of the strong binding between I^−^ and Au [41,42,43]. Based on this, Au nanoparticles labeled with iodine-125 have been prepared and applied in radioactive imaging [42]. Moreover, the interaction force is highly dependent on their surface facets, at a decreasing order of (111) > (110) > (100) [44,45]. By adding I^−^ to inhibit Au deposition on specific facets, Au nanomaterial with various shapes, including nanorods, triangular prisms, spheres, octahedra, and rhombic dodecahedra, have been prepared [46,47,48,49]. Interestingly, it was observed that I^−^ can induce the morphological transformation of Au nanoparticles through the process of Ostwald ripening. During this process, I^−^ at the surface is transformed to iodine atoms via an electron injection process, neutralizing the surface charge and increasing the van der Waals attractive forces among nanoparticles, leading to the formation of aggregates. Potential accumulation because of aggregation induces fragmentation, thereby forming small nanoparticles [43,50]. Although the geometry-dependent localized surface plasmon resonance (LSPR) properties during transformation were investigated by ultraviolet–visible (UV–VIS) absorption spectroscopy and TEM [51,52], the actual mechanism and detailed process remain elusive.

Here, we visually monitored the morphological transformation of single Au nanostars (GNSs) in the presence of I^−^ via DFM under coillumination with red and green lasers. Direct imaging of single nanoparticles revealed the fundamental mechanism of the I^−^-induced shape transformation, which has been developed as a single nanoparticle sensor for I^−^ detection. Lasers with wavelengths of 638 and 534 nm were selected because during their morphological changes to spheres, the LSPR band of the GNSs varied from 638 to 534 nm. As the I^−^ content increased, the scattering spot of the single GNSs underwent color changes from red to orange, yellow, and green. Importantly, this study did not observe aggregation, fusion, and fragmentation, which should occur as per previous studies [43,50]. To assess their morphological changes, the green/red (G/R) intensity ratios were extracted. The ratio was linearly dependent on the I^−^ concentration with a low limit of detection (LOD). The iodide contents in table salt and seaweeds were quantified with good accuracy. Furthermore, X-ray photoemission spectroscopy (XPS) and high-resolution TEM result confirmed that I^−^ adsorption and binding on the GNS surface to form AuI^−^ lowered the energy of its bond with other Au atoms, which facilitated Au atoms from high-energy sites to low-energy ones, inducing the shape transition of GNSs to spherical nanoparticles.

## 2. Materials and Methods

### 2.1. Materials

HAuCl_4_·3H_2_O, (3-aminopropyl)triethoxysilane (APTES), ascorbic acid (AA), and 4-(2-hydroxyethyl)-1-piperazineethanesulfonic acid (HEPES) were purchased from Sigma-Aldrich (St. Louis, MO, USA). Hydrochloric acid (HCl) and nitric acid (HNO_3_) were purchased from Beijing Chemical Reagents Company (Beijing, China). Lead nitrate (Pb(NO_3_)_2_), potassium iodide (KI), sodium acetate trihydrate (CH_3_COONa·3H_2_O), sodium carbonate (Na_2_CO_3_), sodium chloride (NaCl), sodium hydroxide (NaOH), sodium sulfate (Na_2_SO_4_), sodium sulfide (Na_2_S), and sodium thiosulfate (Na_2_S_2_O_3_) were obtained from Sinopharm Chemical Reagent (Shanghai, China). Human SH-SY5Y cells were purchased from the Cell Resource Center of the Institute of Basic Medicine, Chinese Academy of Medical Sciences (Beijing, China). Dulbecco’s Modified Eagle’s Medium (DMEM), fetal bovine serum, and 0.25% pancreatin were acquired from the U.S. Gibco Company (Paisley, UK). Ultrapure water with a resistivity of 18.2 MΩ·cm was produced using a Millipore Milli-Q IQ7003 water purification system. All glassware was cleaned in a bath of freshly prepared aqua regia solution (HCl/HNO_3_ = 3:1 in volume) and thoroughly rinsed with H_2_O before use.

### 2.2. Apparatus

The UV–VIS absorption spectra of the GNSs were obtained using a UV-2700 spectrometer (Shimadzu, Kyoto, Japan). The morphologies of the GNSs were observed using a Titan G2 60-300 microscope (FEI, Thermo Fisher Scientific, Hillsboro, OR, USA). XPS was conducted on a K-alpha multichine surface analyzer from Thermo Scientific (Waltham, MA, USA) with Al Kα radiation as the X-ray source and a pass energy of 100 eV. Zeta potentials were measured using a zeta potential analyzer (Nano-ZS90, Malvern, UK). For DFM imaging, a Nikon Ni-U upright microscope equipped with a 100 W tungsten halogen lamp, an oil immersion dark-field condenser (numerical aperture (NA) = 1.20–1.43), and a 40× Plan Fluor objective lens was used. A DP73 single-chip true-color charge-coupled device (CCD) camera (Olympus, Japan) was mounted on the top of the microscope to capture images. Red (638 nm, 100 mW) and green lasers (534 nm, 100 mW) from Laserland (Wuhan, China) were used to replace the 100 W tungsten halogen lamp for illumination to improve the image quality of the individual GNSs.

### 2.3. Preparation of GNSs

The GNSs were synthesized using a seedless approach according to a previous study [53]. Briefly, 40 mL of the HEPES buffer (75 mmol/L, pH 7.4) was mixed with 59.18 mL of deionized water in a volumetric flask, and then 823 µL of HAuCl_4_ solution (24.28 mmol/L) was added. After shaking for 20–30 s, the solution was left undisturbed in a water bath at 25 °C for 30 min. The solution color slightly changed from light yellow to purple and finally to greenish blue, indicating the successful formation of GNSs. To improve the stability of the GNSs and prolong their storage time, the pH of the solution was adjusted to 9.6 ± 0.1 with a 1 mol/L NaOH solution.

### 2.4. Shape Transition of GNSs Monitored under UV–VIS Spectroscopy

The I^−^-induced morphological variation of GNSs was investigated by UV–VIS spectroscopy. Briefly, aliquots (1 mL) of the GNS solution were added into a 2 mL centrifuge tube, and 1 mL each of the I^−^ solutions at varying concentrations (0, 0.3, 0.5, 1, 1.3, 1.5, 1.6, 1.7, 1.8, 1.9, and 2 µmol/L) was added. After incubation at room temperature for 90 min, the UV–VIS spectra were recorded. Moreover, the time-dependent UV–VIS spectra were also recorded at an interval of 5 min. To confirm whether other anions can have a similar effect, 2 µmol/L of Cl^−^, S_2_O_3_^2−^, OH^−^, SO_4_^2−^, S^2−^, CH_3_COO^−^, CO_3_^2−^, NO^3−^, and AA solutions was added to each GNS solution separately, followed by the recording of their UV–VIS spectra after a 90 min incubation.

### 2.5. Real-Time Monitoring of the Morphological Transformation of Single GNSs Induced by I^−^ under DFM

To anchor GNSs onto the surface, coverslips were cleaned and treated by salinization via soaking in a piranha solution (H_2_SO_4_:H_2_O_2_ = 3:1 in volume) for 1 h. Then, they were extracted and washed with ultrapure water. After drying in a stream of N_2_, the coverslips were placed on a steel shelf and maintained at 105 °C for 2 h to thoroughly remove the water molecules on their surfaces. Subsequently, the coverslips were immersed in ethanol containing 2.5% APTES and left undisturbed for 1 h. Next, they were rinsed twice with ethanol and immersed in ultrapure water for further use.

To monitor the transformation process of single GNSs by DFM, GNSs (10 μL) were cast onto an APTES-modified coverslip surface. After 3 min, the coverslips were rinsed with ultrapure water and reversely placed on a glass slide for further DFM imaging. The images of these GNSs were captured under exposure times of 200, 400, 600, and 800 ms through traditional DFM with a 100 W tungsten lamp. GNSs with 0.5 µmol/L of I^−^ were similarly recorded after incubation for 90 min. To improve image quality, green and red lasers at 534 and 638 nm, respectively, were integrated. After passing through a beam expansion, they were applied as light sources for illumination. The GNSs without and with 0.5 µmol/L of I^−^ were imaged at an exposure time of 50 ms by DFM. The obtained color images of the GNSs were split into red, green, and blue channels using Image J (1.8.0, National Institutes of Health). The G and R intensities of the single GNSs were extracted, and G/R ratios were calculated to assess the morphological changes of the GNSs during their interactions with I^−^.

### 2.6. Sensitive Detection of I^−^ by DFM Imaging of Single GNS Nanoparticles

To investigate the sensitivity, the coverslips loaded with GNSs were soaked separately in a series of KI solutions with various concentrations (e.g., final concentrations of 0, 20, 50, 70, 100, 200, 300, 400, and 500 nM). The GNSs immobilized on the coverslip act as an individual signal-response output sensor, which is not affected by the average value in the homogeneous system and has higher sensitivity. Subsequently, the coverslips were reversely placed on a glass side, and the color images of single nanoparticles were captured through dual-laser DFM. The color DFM images were split into RGB channels using Image J software. The G and R intensities of single GNSs were extracted, and the G/R ratios were calculated to assess I^−^ content.

### 2.7. Selectivity

To investigate the selectivity of our assay toward I^−^, the above procedure was repeated by replacing with other anions, including CO_3_^2−^, S^2−^, OH^−^, S_2_O_3_^2−^, Cl^−^, SO_4_^2−^, NO_3_^−^, CH_3_COO^−^, and AA, followed by capturing their DFM images. All the experiments were repeated three times.

### 2.8. I^−^ Detection in Real Sample

To evaluate the application of our assays, we tested iodine contents in table salts and seaweeds that were bought from a supermarket. In these samples, iodine usually exists in the form of organic iodine, IO_3_^−^, and so on, rather than I^−^. IO_3_^−^ is the main species in table salts; we needed to transform iodine to iodide. Briefly, 1 g of iodized salt was dissolved in 99 mL distilled water, and 1.0 mL of 20 mM AA was added. The mixture solution was heated at 50 °C for 20 min, ensuring that the IO_3_^−^ was reduced to I^−^ [54]. The treatment of seaweeds was according to GB 5009.267-2016 with some modifications. Briefly, 2–3 g of samples was put into a crucible, followed by adding 5 mL of 0.47 M Na_2_CO_3_ solution. Then, the mixture was heated until there was smoke and was placed in a muffle furnace at 600 °C for 4 h and then taken out after the temperature dropped to 200 °C. Subsequently, the ashed sample was transferred to a 100 mL volumetric flask by adding 5 mL water and washing three times. Finally, 1.0 mL of 20 mM AA was added, and the solution was kept at 60 °C for 15 min. If the detected iodide content exceeded the detection range, the sample should be diluted by adding distilled water and redetected. For detection, 750 µL of sample solution was mixed with 750 µL GNS solution (pH 6.8) for 90 min. An amount of 10 µL of solution was added dropwise to the surface of the APTES-modified coverslip and observed under DFM. All analysis results were repeated three times.

## 3. Results and Discussion

### 3.1. Transformation of GNSs after the Introduction of I^−^

**Figure 1:**
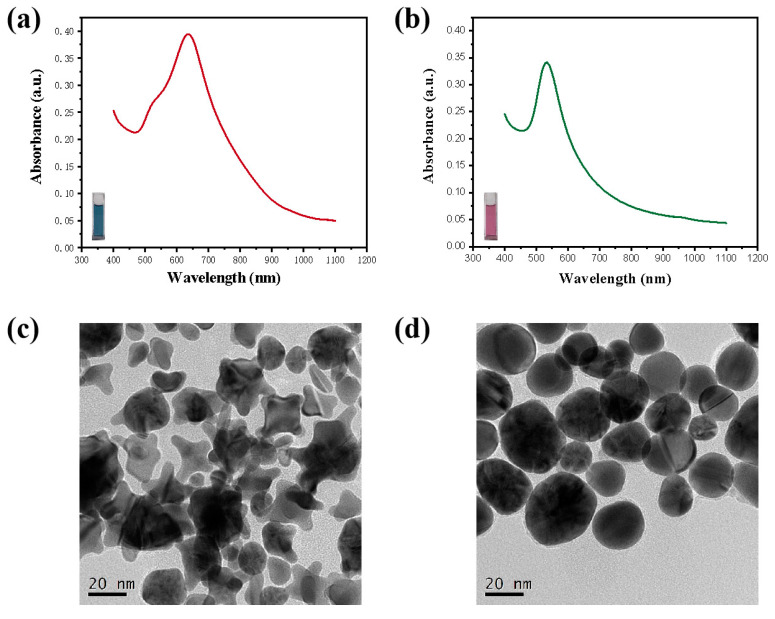
UV–VIS spectra (**a**,**b**) and transmission electron microscopy (TEM) (**c**,**d**) images of GNSs prior to (**a**,**c**) and after (**b**,**d**) incubation with 2 μM iodide for 90 min.

I^−^ can induce the morphological deformation of GNSs, as shown in Figure 1 and as suggested in previous studies [43,50]. Two plasmonic absorption peaks of the GNSs at 520 and 638 nm were assigned to their transverse and longitudinal LSPR bands, respectively (Figure 1a) [53,55,56]. The longitudinal LSPR is closely related to the number, length, and size of the branch [57]. After incubation with I^−^ (2 μM) for 90 min, the GNS solution exhibited one absorption peak at 534 nm (Figure 1b), revealing that the GNSs were transformed into spherical Au nanoparticles. Correspondingly, the solution changed from greenish blue to red. The TEM image (Figure 1c) demonstrated that the as-prepared GNSs had an average diameter of 39.4 ± 1.5 nm with 2–5 branches. After incubation with I^−^, spherical Au nanoparticles with an average diameter of 23.4 ± 0.8 nm were observed (Figure 1d). The time-dependent UV–VIS spectra (Supporting Information Figure S1) of the GNS solution showed that during the transformation, the longitudinal LSPR experienced gradual blue shifts. These results confirmed the shape transition of the GNSs induced by I^−^; however, they did not provide sufficient information to support whether or not the shape changed through the aggregation/fusion/fragmentation processes, as suggested in previous studies [43,50].

### 3.2. Enhanced Image Quality of Single GNSs through DFM with G and R Lasers

**Figure 2:**
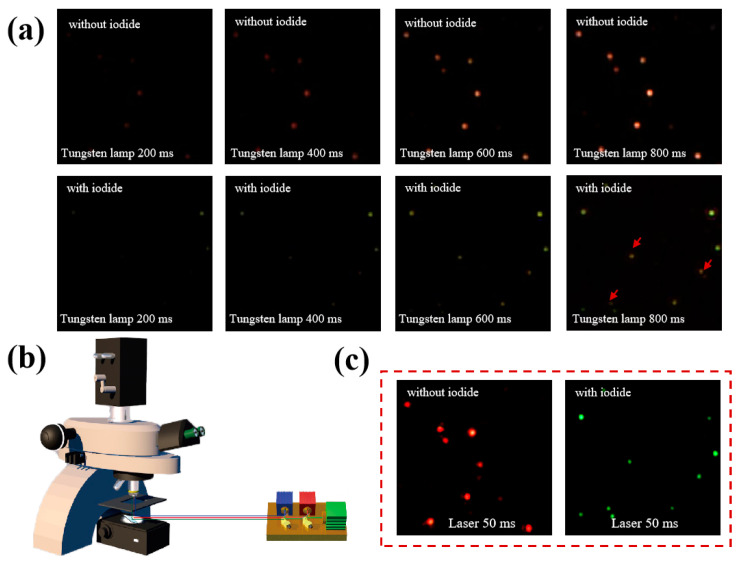
(**a**) DFM images of GNSs without and with incubation with iodide using a 100 W tungsten lamp at various exposure times (200, 400, 600, and 800 ms). (**b**) The scheme of dual-laser DFM. (**c**) DFM images of individual GNSs using R and G laser illumination with an exposure time of 50 ms. The iodide concentration was 2 μM, and the incubation time was 90 min.

Using the conventional DFM with a tungsten lamp (100 W), single GNSs in the absence and presence of 2 µM I^−^ were imaged at various exposure times (200–800 ms) (Figure 2a). It was practically impossible to observe the GNSs under an exposure time of 200 ms. At 400 ms, GNSs exhibited dim red spots. With an increase in exposure time, brightness was greater. However, when the exposure time was raised to 800 ms, multiple spots changed to orange, which should be red according to their longitudinal LSPR. However, after the shape transformation to spheres with the presence of I^−^, dim green spots were visualized with an exposure time at 400 ms. Nanoparticles exhibited green spots at 600 ms, and more yellow-green spots were observed at 800 ms. Based on spectral change in the LSPR from 638 to 534 nm, the color of the nanoparticles should have changed from red to green. The unusual color variations were attributable to two reasons. One is that the wavelength of the tungsten light source covered a range of 360–760 nm, and the light collection efficiency of the CCD was wavelength dependent [58]. The other one is that GNSs had a small size distribution (Figure 1c). Long exposure time changed the saturation, inevitably causing a color change. Furthermore, observing small GNSs at a short exposure time was challenging. Furthermore, the color of nanoparticles recorded under DFM with conventional illumination can hardly reflect the morphological changes. In addition, the long exposure time (>400 ms) required for conventional DFM limited the temporal resolution of GNSs.

To enhance image quality, R and G lasers with wavelengths of 638 and 534 nm were integrated to replace the 100 W tungsten lamp to monitor the deformation of the GNSs induced by I^−^ (Figure 2b). The lasers were selected because of the LSPR variation during the transformation. At a short exposure time of 50 ms, the color of all GNSs was bright red, and after the transformation, it was green (Figure 2c). The improved imaging quality was attributed to the merits of lasers, including their strong light intensity and monochromatic properties [58]. Additionally, a short exposure time was beneficial for better temporal resolution. Moreover, because only G and R lasers were employed, the scattering spots were in the clear-cut color gamut comprising red and green. Because of the wavelength of light scattering, the color variations are highly correlated with morphological change. Therefore, the R and G laser illuminations are effective for observing small nanoparticles in the transformation process.

### 3.3. Monitoring the Dynamic Transformation of Single GNSs

**Figure 3:**
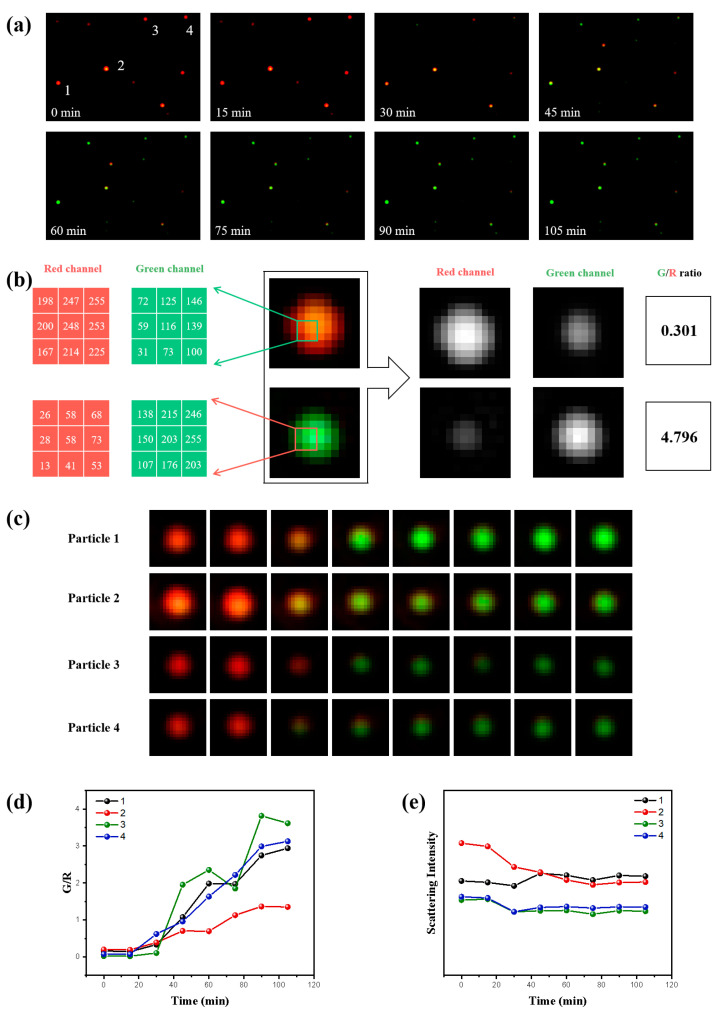
(**a**) Real-time monitoring of GNSs during the shape transformation induced by 0.5 μM iodide at an exposure time of 50 ms (the images were captured at 0, 15, 30, 45, 60, 75, 90, and 105 min). (**b**) R and G channel images of one selected GNS prior to and after transformation induced by iodide. (**c**) Images, (**d**) G/R ratio, and (**e**) scattering intensity of four selected GNSs.

Figure 3a shows that after adding I^−^ (0.5 µM), the red GNS spots gradually changed to orange, yellow, and green, revealing that I^−^ induced the dynamic morphological changes. After 30 min, 33.5% of the spots turned orange. When reaction time was prolonged to 90 min, practically all nanoparticles became green. The results showed inhomogeneous reaction rates among different GNSs, suggesting that reactions, like that between small molecules, follow kinetic models. In addition, a few particles appeared after incubation since the particles suspended in the channel were then adsorbed onto the slide. To quantitatively weigh the changes, we split the color images into RGB channels, and the G and R channel intensities of single GNSs were extracted [23,58]. Figure 3b shows images of the GNSs in the R and G channel, displaying evident changes in their intensities after transformation. Therefore, it is feasible to use G/R ratios to record the reaction kinetic. To check whether G/R ratios are identical to spectral change, we placed a transmission grating beam splitter (TGBS, 70 lines/mm) in the light collection path to acquire the scattering spectrum of the single GNSs [59]. Results showed that the changes in the G/R ratio obtained by splitting images with/without the TGBS were identical (Supporting Information Figure S2). More importantly, during the process, diverse behaviors were observed. Figure 3c,d shows four representative examples. For particle 1, the G/R ratio started to increase at 20 min, became fast within 30–60 min, turned slow during 60–75 min, and became fast again then. For particle 2, the G/R ratio generally increased within 0–100 min. Interestingly, the G/R ratio of particle 3 increased during 30–60 min, slightly declined within 60–75 min, and rose to the maximum, exhibiting variable reaction rates. Particle 4 exhibited a continuous ascending G/R ratio over 15–90 min. Fascinatingly, during transformation, the scattering intensities of particles 2–4 decreased, and that for particle 1 increased (Figure 3e). The diverse kinetics among various GNSs occurring among nanoparticles was probably due to the difference in the sizes and shapes as well as active sites and surface energies. Above all, the GNSs underwent shape transformations individually, without aggregation and fragmentation.

### 3.4. Sensitivity and Selectivity

**Figure 4:**
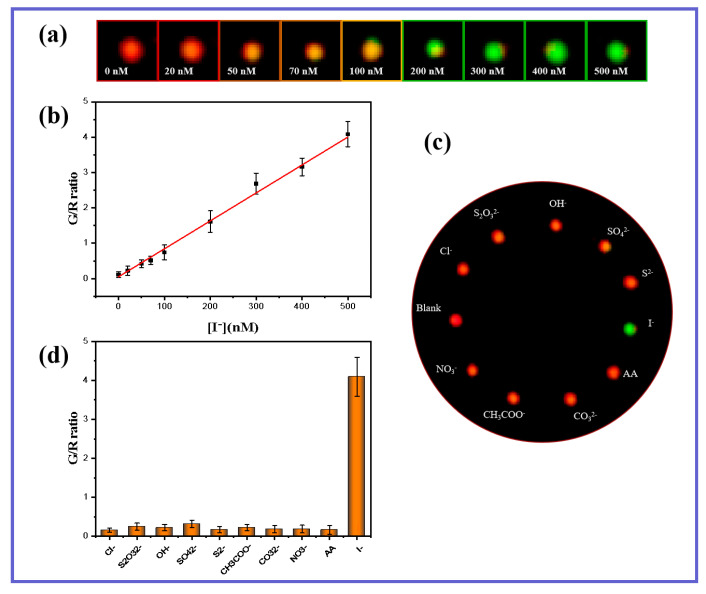
(**a**) Scattering images of GNSs in the presence of iodide within the concentration range of 0–500 nM. (**b**) G/R ratio vs. iodide concentration. (**c**) Images and (**d**) G/R ratio of GNSs with an exposure of 0.5 µM of other anions, including Cl^−^, S_2_O_3_^2−^, OH^−^, SO_4_^2−^, S^2−^, CH_3_COO^−^, CO_3_^2−^, NO_3_^−^, and AA.

The scattering images of single GNSs at increasing concentrations (0–500 nM) of I^−^ were recorded (Figure 4a) and demonstrated concentration-dependent kinetics. With an increase in the I^−^ concentration, single GNSs turned from red to orange, yellow, and green. The color change in the single GNSs was effortlessly distinguished under microscopy when the I^−^ concentration exceeded 50 nM. Note that such a color change was due to the direct interaction between I^−^ and GNRs without the engagement of other reagents, indicating an assay with high convenience and practicality. To quantify I^−^, G/R ratios were statistically calculated from the intensities of 150 single GNSs. A linear relationship was gained between the G/R ratio and I^−^ concentration (Figure 4b). With I^−^ concentration over the range of 0–500 nM, a linear equation was constructed: G/R = 0.054 + 0.0079 × C_iodide_ with a correlation coefficient (R^2^) of 0.993. Owing to the high anisotropy of the GNSs, the Au atoms at the edges exhibited high chemical activity. Thus, they interacted relatively readily with I^−^. Only with additional I^−^, the Au atoms with low chemical activities can interact with I^−^. It is reasonable that Au atoms on the nanoparticle surface are not in the complete lattice; thus, they have few neighbors and exhibit high chemical activity. The LOD was 6.9 nM (LOD = 3σ/slope, where σ is the standard deviation of five blank samples), which is more sensitive than the reported fluorescence and colorimetric methods (Table 1).

Other anions, such as Cl^−^ and S_2_O_3_^2−^, failed to induce the GNS shape transformation (Figure 4c,d), thus suggesting minimum interference. Dissimilar to I^−^, other anions, including Cl^−^, S_2_O_3_^2−^, OH^−^, SO_4_^2−^, S^2−^, CH_3_COO^−^, CO_3_^2−^, NO_3_^−^, and AA, cannot induce GNS shape deformation, as also confirmed in the UV–VIS absorption spectra (Supporting Information Figure S3). S^2−^ and S_2_O_3_^2−^ exhibited high affinity to Au [60,61], and the solubility product constant values for Au_2_S and Au_2_S_2_O_3_ were 1.6 × 10^−73^ and 3.2 × 10^−27^, whereas that for AuI^−^ was 1.6 × 10^−23^ [62,63]. Although the adsorption of S^2−^ and S_2_O_3_^2−^ on the Au surface was more substantial than I^−^, they could not trigger the shape transformation of the GNSs. We can infer that the morphological change was because of not only the strong affinity to Au but also specific properties of the AuI^−^ complexes after being adsorbed onto the surface.

**Table 1:** Comparison of various iodide detection methods.

Method	Linear Range	LOD	Ref
Colorimetric	10–600 nM	10 nM	[64]
Fluorescence	0.5–20 μM	430 nM	[65]
Fluorescence	0.1–6 μM	90 nM	[66]
Colorimetric	8.8–260 nM	8.8 nM	[51]
Fluorescence	0–200 μM	22.6 nM	[67]
Fluorescence	0–90 μM	92.3 nM	[68]
Single-particle color imaging	0–500 nM	6.9 nM	This work

### 3.5. Mechanism for I^−^-Inducing Transformation of GNSs

**Figure 5:**
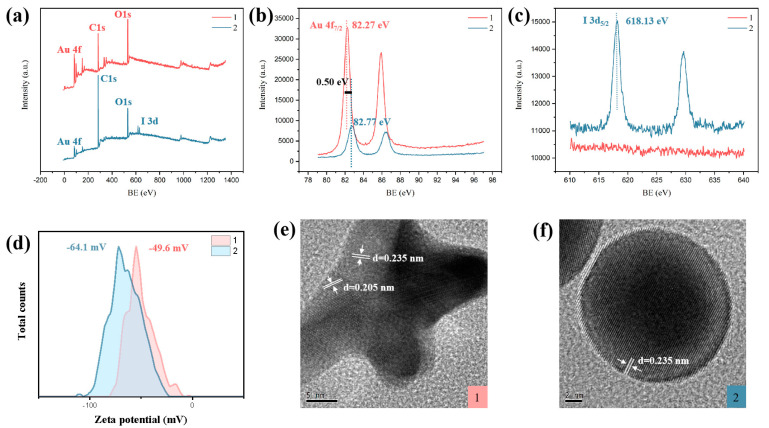
XPS survey spectra of GNSs (**a**), Au 4f (**b**), and I 3d (**c**), Zeta potentials (**d**), and HRTEM images (**e**,**f**) of GNSs before (1) and after (2) incubation with 2 µM iodide for 90 min.

**Scheme 1:**
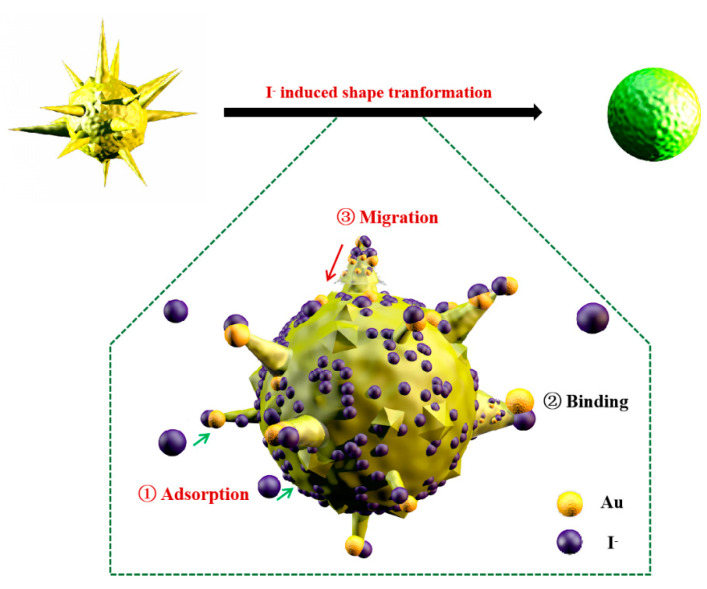
The mechanism for the shape transformation of GNSs induced by iodide.

According to previous studies [41,43], the morphological transformation of Au nanoparticles induced by I^−^ involved aggregation, fusion, and fragmentation through the Ostwald ripening process based on TEM images and UV–VIS spectra. However, in this study, we observed that the transformation from GNSs to spherical nanoparticles was a self-fusion process through adsorption, binding, and migration (Scheme 1). We propose that I^−^ can be spontaneously absorbed on the surface, and the formation of AuI^−^ enabled the migration of Au atoms from high-energy sites to low-energy sites. To support this hypothesis, we recorded the XPS spectra of GNSs in the presence and absence of 2.0 µM I^−^, as shown in Figure 5. A new peak at 618 eV was responsible for the surface I^−^ (Figure 5a). The binding energy (BE) for Au 4f_7/2_ of the GNSs located at 82.27 eV shifted to 82.77 eV after its interaction with I^−^, thus supporting their interaction without alternating the oxidation state of Au (Figure 5b) [69]. After being absorbed, the BE for I 3d_5/2_ located at 618.13 eV indicated the existence of negative valence of I^−^ (Figure 5c) [70]. The zeta potential of the GNSs decreased from −49.6 to −64.1 mV after incubation with I^−^, indicating the replacement of surface molecule HEPES by I^−^ [71]. High-resolution TEM images of the GNSs without and with 2 μM of I^−^ were taken (Figure 5e,f). Lattice spacings of 0.235 and 0.205 nm were observed on the GNSs, representing typical (111) and (200) crystal facets, respectively. However, after being transformed to spherical Au nanoparticles, only (111) was observed. I^−^ did not enter the interior of the Au nanoparticles. Moreover, the deformed spherical nanoparticles were particularly round without edges or corners.

To examine whether the transformation was not attributed to the electron injection process, we added extra NaBH_4_ and starch to the reacting solution to examine whether I_2_ was formed (Supporting Information Figure S4). Owing to the shape transformation, the GNS solution changed from greenish blue to red after adding I^−^. In the presence of 0.1 mM of NaBH_4_ with a strong reducing ability to prevent the formation of iodine, the GNSs still underwent a shape change, revealing that iodine was not responsible for the shape transformation. I_2_ can interact with starch to form blue complexes, but no change was observed on the UV–VIS curve of GNSs with the presence of extra starch, thus again confirming that iodine was not formed. Additionally, in the absence of light, 2 µM of I^−^ still induced GNS transformation, ruling out the essential role of photo irradiation in the process.

Single nanoparticle imaging directly confirmed that aggregation, fusion, and fragmentation did not occur during the transformation of GNSs to spherical Au nanoparticles. To investigate whether other shaped nanoparticles experienced similar changes, Au nanorods, Au nanoprisms, and large GNSs (~100 nm, capped by citrate) were prepared as per previous studies with slight modifications (Supporting Information Figure S5) [72,73]. The results showed that 2 µM of I only induced the transformation of the large GNSs. Thus, the specific shape of GNSs with various corners and branches was critical for the shape deformation, for the existence of highly active Au atoms on the edges of the GNSs. The GNSs capped with citrate and HEPES underwent transformation, suggesting that the surface capping agents did not account for the shape transformation. The growth of large spherical Au nanoparticles was not observed in this study. Therefore, we ruled out the occurrence of Ostwald ripening [74,75]. Our results are consistent with a previous study in that the chemical adsorption of I^−^ onto the GNSs played an important role in nanoparticle transformation [76]. However, it was not sufficient, and the AuI^−^ formation took effect for shape transition because other ions such as S^2−^ with strong chemical adsorption could not induce this phenomenon. Thermodynamically, I^−^ induced the shape transformation in a spontaneous process, which was primarily because spherical Au nanoparticles were more energetically favored than star-shaped particles. Moreover, the Au atoms on the nanoparticle surface were energetically less stable than those in the interior because all atoms inside bonded to 12 neighbors, while the atoms on the surface were bound to fewer. Therefore, the mechanism for the transformation was that I^−^ adsorbed onto the surface of the GNSs was bound to an Au atom to form AuI^−^, lowering the energy of its bond with other Au atoms, which facilitated the diffusion of this atom across the nanoparticle surface to low-energy sites.

### 3.6. Single GNS Sensor for the Quantification of I^−^ Real Sample Detection

To promote the application of this probe and method in real sample detection, table salt and seaweeds were used to monitor the I^−^ content. For table salts, we transformed iodine in salts to iodide. AA was also effective in reducing IO_3_^−^ to I^−^. The treatment of seaweeds and a complex vitamin tablet was according to GB 5009.267-2016 with some modifications. Considering the high sensitivity of our assay, the seaweeds were diluted 1000 times using distilled water. For detection, 750 µL of sample solution was mixed with 750 µL GNS solution (pH 6.8) for 90 min. An amount of 10 µL of the solution was dropped onto the surface of the APTES-modified coverslip and observed under DFM. The iodide contents in table salt, seaweeds, and complex vitamin tablets were precisely quantified, as shown in Table 2. The iodized salt contained 22.8 mg/kg of iodide, meeting the requirement of the standard. The iodide content in seaweeds was as high as 1620.4 mg/kg. In addition, the sample recoveries varied from 98.5% to 106.5%, and the relative standard deviation (RSD) was in the range of 3.2%–6.7%, which verified the reliability of the GNSs for real complex biological samples.

## 4. Conclusions

Here, DFM with R and G laser illumination afforded high-quality scattering images of single GNSs, thus providing detailed information during the shape transformation of GNSs induced by I^−^. With increasing I^−^ concentration, the color of the single nanoparticles changed from red to orange, yellow, and green. The G/R ratios reflected the degree of morphological transformation, which was dependent on the I^−^ concentration. A single nanoparticle sensor was developed for I^−^ quantification with a LOD of 6.9 nM. Importantly, the I^−^ contents in table salt, seaweeds, and a complex vitamin tablet were quantified with good accuracy. The processes of aggregation, fusion, and fragmentation did not occur, dissimilar to the results of previous reports. The I^−^-induced transformation of Au nanoparticles was investigated by XPS, high-resolution TEM, and zeta potential measurements. It confirmed that the valence of I^−^ did not change during the transformation process, and Ostwald ripening was not responsible for shape transition. The strong absorption and binding of I^−^ onto the Au surface were critical for shape transformation. However, the specific properties of AuI^−^ played more critical roles, because the interaction of I^−^ with the Au atom lowered the energy of its bond with other Au atoms, which facilitated the diffusion of this atom across the nanoparticle surface to low-energy sites.

## Figures and Tables

**Table 2:** Detection of iodide content in table salts and seaweeds (*n* = 3).

Sample	Added (mg/kg)	Detected (mg/kg)	Recovery (%)	RSD (%)
Uniodized table salt	0	ND	/	/
Table salt	0	22.8	/	3.2
	5	28.1	101.3	4.5
	10	33.9	104.8	3.8
	20	44.3	106.5	3.6
Seaweeds (chilled)	0	1620.4	/	3.7
	100	1721.4	100.1	5.2
	200	1805.4	98.5	6.7
	500	2115.4	99.4	5.9

Note: ND denotes not detected.

## Data Availability

The data presented in this study are available upon reasonable request from the corresponding author.

## Supplementary Materials

The following supporting information can be downloaded at: https://www.mdpi.com/article/10.3390/nano12152555/s1, Figure S1: The UV–VIS spectra of GNS solution in the presence of 2 μM KI after various periods (0–90 min) of reaction time; Figure S2: Representative G/R ratios and scattering spectra of single GNSs irradiated with R and G lasers. Exposure time: 50 ms; reaction time: 0, 30, 60, and 90 min. Inset: Corresponding DFM images of single GNSs after treatment with 0.5 µM KI, showing a color change from red to green; Figure S3: UV–VIS spectra of the selective response of GNSs with the presence of different anions, including Cl^−^, S_2_O_3_^2−^, OH^−^, SO_4_^2−^, S^2−^, CH_3_COO^−^, CO_3_^2−^, NO^3−^, and AA; Figure S4: Photographs and UV–VIS spectra of GNSs (A) and in the presence of 2 µM KI (B), 2 µM KI and 100 µM NaBH_4_ (C),100 μM I_2_ that had reacted with 0.5% starch for 3 min (D), and 2 µM KI and 0.5% starch (E); Figure S5: UV–VIS spectra and DFM images of Au nanorods (A), Au nanoprisms (B), and larger GNSs capped with citrate (C) in the presence of 2 μM KI.

## References

[1] Xu S., Yu X., Chen Z., Zeng Y., Guo L., Li L., Luo F., Wang J., Qiu B., Lin Z. (2020). Real-Time Visualization of the Single-Nanoparticle Electrocatalytic Hydrogen Generation Process and Activity under Dark Field Microscopy. Anal. Chem..

[2] Zhang H.Z., Li R.S., Gao P.F., Wang N., Lei G., Huang C.Z., Wang J. (2017). Real-Time Dark-Field Light Scattering Imaging to Monitor the Coupling Reaction with Gold Nanorods as an Optical Probe. Nanoscale.

[3] Wang H., Zhao W., Zhao Y., Xu C.-H., Xu J.-J., Chen H.-Y. (2020). Real-Time Tracking The Electrochemical Synthesis of Au@Metal Core-Shell Nanoparticles toward Photo Enhanced Methanol Oxidation. Anal. Chem..

[4] Chen M.-M., Xu C.-H., Zhao W., Chen H.-Y., Xu J.-J. (2021). Super-Resolution Electrogenerated Chemiluminescence Microscopy for Single-Nanocatalyst Imaging. J. Am. Chem. Soc..

[5] Lei G., Gao P.F., Yang T., Zhou J., Zhang H.Z., Sun S.S., Gao M.X., Huang C.Z. (2017). Photoinduced Electron Transfer Process Visualized on Single Silver Nanoparticles. ACS Nano.

[6] Khelfa A., Nelayah J., Amara H., Wang G., Ricolleau C., Alloyeau D. (2021). Quantitative In Situ Visualization of Thermal Effects on the Formation of Gold Nanocrystals in Solution. Adv. Mater..

[7] Ge F., Xue J., Du Y., He Y. (2021). Unmodified Single Nanoparticles Undergo a Motion-Pattern Transition on the Plasma Membrane before Cellular Uptake. Nano Today.

[8] Liu M., Wang F., Zhang X., Mao X., Wang L., Tian Y., Fan C., Li Q. (2020). Tracking Endocytosis and Intracellular Distribution of Spherical Nucleic Acids with Correlative Single-Cell Imaging. Nat. Protoc..

[9] Wei Z., Qi H., Li M., Tang B., Zhang Z., Han R., Wang J., Zhao Y. (2012). Watching Single Gold Nanorods Grow. Small.

[10] Zhao Y., Kong X., Shearer M.J., Ding F., Jin S. (2021). Chemical Etching of Screw Dislocated Transition Metal Dichalcogenides. Nano Lett..

[11] Furuya R., Omagari S., Tan Q., Lokstein H., Vacha M. (2021). Enhancement of the Photocurrent of a Single Photosystem I Complex by the Localized Plasmon of a Gold Nanorod. J. Am. Chem. Soc..

[12] Jiang Y., Zhu G., Lin F., Zhang H., Jin C., Yuan J., Yang D., Zhang Z. (2014). In Situ Study of Oxidative Etching of Palladium Nanocrystals by Liquid Cell Electron Microscopy. Nano Lett..

[13] Liu Y., Lin X.M., Sun Y., Rajh T. (2013). In Situ Visualization of Self-Assembly of Charged Gold Nanoparticles. J. Am. Chem. Soc..

[14] Chee S.W., Tan S.F., Baraissov Z., Bosman M., Mirsaidov U. (2017). Direct Qbservation of the Nanoscale Kirkendall Effect during Galvanic Replacement Reactions. Nat. Commun..

[15] Arrigo R., Sasaki T., Callison J., Gianoliob D., Schuster M.E. (2022). Monitoring Dynamics of Defects and Single Fe Atoms in N-Functionalized Few-Layer Graphene by In Situ Temperature Programmed Scanning Transmission Electron Microscopy. J. Energ. Chem..

[16] Dachraoui W., Keller D., Henninen T.R., Ashton O.J., Erni R. (2021). Atomic Mechanisms of Nanocrystallization via Cluster-Clouds in Solution Studied by Liquid-Phase Scanning Transmission Electron Microscopy. Nano Lett..

[17] Zhang X., Wang H., Wang H., Zhang Q., Xie J., Tian Y., Wang J., Xie Y. (2014). Single-Layered Graphitic-C3n4 Quantum Dots for Two-Photon Fluorescence Imaging of Cellular Nucleus. Adv. Mater..

[18] Shang L., Doerlich R.M., Brandholt S., Schneider R., Trouillet V., Bruns M., Gerthsen D., Nienhaus G.U. (2011). Facile Preparation of Water-Soluble Fluorescent Gold Nanoclusters for Cellular Imaging Applications. Nanoscale.

[19] Mehta S.B., Mcquilken M., La Riviere P.J., Tani T. (2016). Dissection of Molecular Assembly Dynamics by Tracking Orientation and Position of Single Molecules in Live Cells. Proc. Natl. Acad. Sci. USA.

[20] Wang K., Li S., Liu Y., Jiang L., Zhang F., Wei Y., Zhang Y., Qi Z., Wang K., Liu S. (2017). In Situ Detection and Imaging of Telomerase Activity in Cancer Cell Lines via Disassembly of Plasmonic Core-Satellites Nanostructured Probe. Anal. Chem..

[21] Saqib M., Fan Y., Hao R., Zhang B. (2021). Optical Imaging of Nanoscale Electrochemical Interfaces in Energy Applications. Nano Energy.

[22] Hwu S., Blickenstorfer Y., Tiefenauer R.F., Gonnelli C., Schmidheini L., Lüchtefeld I., Hoogenberg B.-J., Gisiger A.B., Vörös J. (2019). Dark-Field Microwells toward High-Throughput Direct Mirna Sensing with Gold Nanoparticles. ACS Sens..

[23] Hao J., Xiong B., Cheng X.D., He Y., Yeung E.S. (2014). High-Throughput Sulfide Sensing with Colorimetric Analysis of Single Au-Ag Core-Shell Nanoparticles. Anal. Chem..

[24] Zhao Y., Zhao W., Chen H.-Y., Xu J.-J. (2021). Dark-Field Microscopic Real-Time Monitoring the Growth of Au On Cu2o Nanocubes for Ultra-Sensitive Glucose Detection. Anal. Chim. Acta.

[25] Chuang C.C., Chang C.W. (2015). Complexation of Bioreducible Cationic Polymers with Gold Nanoparticles for Improving Stability in Serum and Application on Nonviral Gene Delivery. ACS Appl. Mater. Interfaces.

[26] Shi L., Jing C., Ma W., Li D.-W., Halls J.E., Marken F., Long Y.-T. (2013). Plasmon Resonance Scattering Spectroscopy at the Single-Nanoparticle Level: Real-Time Monitoring of a Click Reaction. Angew. Chem. Int. Ed..

[27] Novo C., Funston A.M., Mulvaney P. (2008). Direct Observation of Chemical Reactions on Single Gold Nanocrystals Using Surface Plasmon Spectroscopy. Nat. Nanotechnol..

[28] Novo C., Funston A.M., Gooding A.K., Mulvaney P. (2009). Electrochemical Charging of Single Gold Nanorods. J. Am. Chem. Soc..

[29] Xie T., Jing C., Ma W., Ding Z., Gross A.J., Long Y.-T. (2014). Real-Time Monitoring for the Morphological Variations of Single Gold Nanorods. Nanoscale.

[30] Liu Y., Huang C.Z. (2013). Real-Time Dark-Field Scattering Microscopic Monitoring of the In Situ Growth of Single Ag@Hg Nanoalloys. ACS Nano.

[31] Schopf C., Wahl A., Martin A., O’Riordan A., Iacopino D. (2016). Direct Observation of Mercury Amalgamation on Individual Gold Nanorods Using Spectroelectrochemistry. J. Phys. Chem. C.

[32] Smith J.G., Yang Q., Jain P.K. (2014). Identification of a Critical Intermediate in Galvanic Exchange Reactions by Single-Nanoparticle-Resolved Kinetics. Angew. Chem.-Int. Ed..

[33] Zhou J., Yang T., He W., Pan Z.Y., Huang C.Z. (2018). A Galvanic Exchange Process Visualized on Single Silver Nanoparticles Via Dark-Field Microscopy Imaging. Nanoscale.

[34] Ahad F., Ganie S.A. (2010). Iodine Metabolism and Iodine Deficiency Disorders Revisited. Indian J. Endocrinol. Metab..

[35] Zimmermann M.B., Boelaert K. (2015). Iodine Deficiency and Thyroid Disorders. Lancet Diabetes Endocrinol..

[36] Leung A.M., Braverman L.E. (2012). Iodine-Induced Thyroid Dysfunction. Curr. Opin. Endocrinol. Diabetes Obes..

[37] Carl A., Krejbjerg A., Laurberg P. (2014). Epidemiology of Nodular Goitre. Influence of Iodine Intake. Best Pract. Res. Clin. Endocrinol. Metab..

[38] Laurberg P., Cerqueira C., Ovesen L., Rasmussen L.B., Perrild H., Andersen S., Pedersen I.B., Carlé A. (2010). Iodine Intake as a Determinant of Thyroid Disorders in Populations. Best Pract. Res. Clin. Endocrinol. Metab..

[39] Clark M.N. (1981). A Fatal Case of Iodine Poisoning. Clin. Toxicol..

[40] Pennington J.A. (1990). A Review of Iodine Toxicity Reports. J. Am. Diet. Assoc..

[41] Wang J., Li Y.F., Huang C.Z. (2008). Identification of Iodine-Induced Morphological Transformation of Gold Nanorods. J. Phys. Chem. C.

[42] Walsh A.A. (2017). Chemisorption of Iodine-125 to Gold Nanoparticles Allows for Real-Time Quantitation and Potential Use in Nanomedicine. J. Nanopart. Res..

[43] Cheng W., Dong S., Wang E. (2003). Iodine-Induced Gold-Nanoparticle Fusion/Fragmentation/Aggregation and Iodine-Linked Nanostructured Assemblies on a Glass Substrate. Angew. Chem. Int. Ed..

[44] Ha T.H., Koo H.J., Chung B.H. (2007). Shape-Controlled Syntheses of Gold Nanoprisms and Nanorods Influenced by Specific Adsorption of Halide Ions. J. Phys. Chem. C.

[45] Xiang Y., Wu X., Liu D., Feng L., Zhang K., Chu W., Zhou W., Xie S. (2008). Tuning the Morphology of Gold Nanocrystals by Switching the Growth Of {110} Facets from Restriction to Preference. J. Phys. Chem. C.

[46] Jessl S., Tebbe M., Guerrini L., Fery A., Alvarez-Puebla R.A., Pazos-Perez N. (2018). Silver-Assisted Synthesis of Gold Nanorods: The Relation Between Silver Additive and Iodide Impurities. Small.

[47] Millstone J.E., Wei W., Jones M.R., Yoo H., Mirkin C.A. (2008). Iodide Ions Control Seed-Mediated Growth of Anisotropic Gold Nanoparticles. Nano Lett..

[48] Chung P.-J., Lyu L.-M., Huang M.H. (2011). Seed-Mediated and Iodide-Assisted Synthesis of Gold Nanocrystals with Systematic Shape Evolution from Rhombic Dodecahedral to Octahedral Structures. Chem.-Eur. J..

[49] Grzelczak M., SNchez-Iglesias A., Rodríguez-González B., Alvarez-Puebla R., Pérez-Juste J., Liz-Marzán L.M. (2008). Influence of Iodide Ions on the Growth of Gold Nanorods: Tuning Tip Curvature and Surface Plasmon Resonance. Adv. Funct. Mater..

[50] Zhang J., Xu X., Yang C., Yang F., Yang X. (2011). Colorimetric Iodide Recognition and Sensing by Citrate-Stabilized Core/Shell Cu@Au Nanoparticles. Anal. Chem..

[51] Yang X.-H., Ling J., Peng J., Cao Q.-E., Ding Z.-T., Bian L.-C. (2013). A Colorimetric Method for Highly Sensitive and Accurate Detection of Iodide by Finding the Critical Color in a Color Change Process Using Silver Triangular Nanoplates. Anal. Chim. Acta.

[52] Zhen S., Wu T., Huang X., Li Y., Huang C. (2016). Facile Synthesis of Gold Nanoflowers as Sers Substrates and Their Morphological Transformation Induced by Iodide Ions. Sci. China Chem..

[53] Xie J., Lee J.Y., Wang D.I.C. (2007). Seedless, Surfactantless, High-Yield Synthesis of Branched Gold Nanocrystals in Hepes Buffer Solution. Chem. Mater..

[54] Machado A., Mesquita R.B., Oliveira S., Bordalo A.A. (2017). Development of a Robust, Fast Screening Method for the Potentiometric Determination of Iodide in Urine and Salt Samples. Talanta.

[55] Liu X.-L., Wang J.-H., Liang S., Yang D.-J., Nan F., Ding S.-J., Zhou L., Hao Z.-H., Wang Q.-Q. (2014). Tuning Plasmon Resonance of Gold Nanostars for Enhancements of Nonlinear Optical Response and Raman Scattering. J. Phys. Chem. C.

[56] Dam D.H.M., Lee J.H., Sisco P.N., Co D.T., Zhang M., Wasielewski M.R., Odom T.W. (2012). Direct Observation of Nanoparticle-Cancer Cell Nucleus Interactions. ACS Nano.

[57] Liu Y., Huang C.Z. (2013). Screening Sensitive Nanosensors Via The Investigation of Shape-Dependent Localized Surface Plasmon Resonance of Single Ag Nanoparticles. Nanoscale.

[58] Cheng X., Dai D., Yuan Z., Peng L., He Y., Yeung E.S. (2014). Color Difference Amplification between Gold Nanoparticles in Colorimetric Analysis with Actively Controlled Multiband Illumination. Anal. Chem..

[59] Cheng J., Liu Y., Cheng X., He Y., Yeung E.S. (2010). Real Time Observation of Chemical Reactions of Individual Metal Nanoparticles with High-Throughput Single Molecule Spectral Microscopy. Anal. Chem..

[60] Lezna R.O., Tacconi N., Arvia A.J. (1990). Modulated Reflectance Spectroscopy and Voltammetry of the Sulphide/Gold System. J. Electroanal. Chem..

[61] Lay M., Varazo K., Stickney J.L. (2003). Formation of Sulfur Atomic Layers on Gold from Aqueous Solutions of Sulfide and Thiosulfate: Studies Using Ec-Stm, Uhv-Ec, and Tlec. Langmuir.

[62] Fan J., Li R., Xu P., Di J., Tu Y., Yan J. (2014). Sensitive Sulfide Sensor with a Trypsin-Stabilized Gold Nanocluster. Anal. Sci..

[63] Aylmore M.G., Muir D.M. (2001). Thiosulfate Leaching of Gold—A Review. Miner. Eng..

[64] Chen L., Lu W., Wang X., Chen L. (2013). A Highly Selective and Sensitive Colorimetric Sensor for Iodide Detection Based on Anti-Aggregation of Gold Nanoparticles. Sens. Actuators B Chem..

[65] Du F., Zeng F., Ming Y., Wu S. (2013). Carbon Dots-Based Fluorescent Probes for Sensitive and Selective Detection of Iodide. Microchim. Acta.

[66] Nabavi S., Alizadeh N. (2014). A Highly Sensitive and Selective Turn-on Fluorescence Sensor for Iodide Detection Based on Newly Synthesized Oligopyrrole Derivative and Application to Real Samples. Sens. Actuators B Chem..

[67] Zhang R.X., Li P.F., Zhang W.J., Li N., Zhao N. (2016). A Highly Sensitive Fluorescent Sensor with Aggregation-Induced Emission Characteristics for the Detection of Iodide and Mercury Ions in Aqueous Solution. J. Mater. Chem. C.

[68] Huang H., Weng Y., Zheng L., Yao B., Weng W., Lin X. (2017). Nitrogen-Doped Carbon Quantum Dots as Fluorescent Probe for “off-on” Detection of Mercury Ions, L-Cysteine and Iodide Ions. J. Colloid Interface Sci..

[69] Bravo B.G., Michelhaugh S.L., Soriaga M.P., Villegas I., Suggs D.W., Stickney J.L. (1991). Anodic Underpotential Deposition and Cathodic Stripping of Iodine at Polycrystalline and Single-Crystal Gold. Studies by Leed, Aes, Xps, and Electrochemistry. J. Phys. Chem..

[70] Li K., Zhao Y., Zhang P., He C., Deng J., Ding S., Shi W. (2016). Combined Dft and Xps Investigation of Iodine Anions Adsorption on the Sulfur Terminated (001) Chalcopyrite Surface. Appl. Surf. Sci..

[71] Xi W., Haes A.J. (2019). Elucidation of Hepes Affinity to and Structure on Gold Nanostars. J. Am. Chem. Soc..

[72] Ni W., Kou X., Yang Z., Wang J. (2008). Tailoring Longitudinal Surface Plasmon Wavelengths, Scattering and Absorption Cross Sections of Gold Nanorods. ACS Nano.

[73] Ren W., Huang Z., Xu Y., Li Y., Ji Y., Su B. (2015). Urchin-Like Gold Nanoparticle-based Immunochromatographic Strip Test for Rapid Detection of Fumonisin B-1 in Grains. Anal. Bioanal. Chem..

[74] Liu B., Zeng H. (2010). Symmetric and Asymmetric Ostwald Ripening in the Fabrication of Homogeneous Core–Shell Semiconductors. Small.

[75] Redmond P.L., Hallock A.J., Brus L.E. (2005). Electrochemical Ostwald Ripening of Colloidal Ag Particles on Conductive Substrates. Nano Lett..

[76] Sun M., Ran G., Fu Q., Xu W. (2015). The Effect of Iodide on the Synthesis of Gold Nanoprisms. J. Exp. Nanosci..

