# Supplementary material for: In Situ Direct Monitoring of the Morphological Transformation of Single Au Nanostars Induced by Iodide through Dual-Laser Dark-Field Microscopy: Unexpected Mechanism and Sensing Applications
Source: Nanomaterials (Basel). 2022 Jul 25;12(15):2555. doi: 10.3390/nano12152555 (PMC9330405; doi:10.3390/nano12152555)
Supplement: Supplementary file 1 [file nanomaterials-12-02555-s001.zip › nanomaterials-1812606-supplementary.pdf]

Supplementary Materials

In Situ Direct Monitoring of the Morphological Transformation of Single Au Nanostars Induced by Iodide through Dual-Laser Dark-Field Microscopy: Unexpected Mechanism and Sensing Applications

Weizhen Xu, Hongmei Luo, Min Ouyang, Tiantian Long and Qinlu Lin *

National Engineering Laboratory for Rice and By-Products Further Processing, College of Food Science and Engineering, Central South University of Forestry & Technology, Changsha 410004, China; 20180100023@csuft.edu.cn (W.X.); 20201200556@csuft.edu.cn (H.L.); 20201200547@csuft.edu.cn (M.O.); 20191200447@csuft.edu.cn (T.L.)

* Correspondence: LinQL0403@163.com

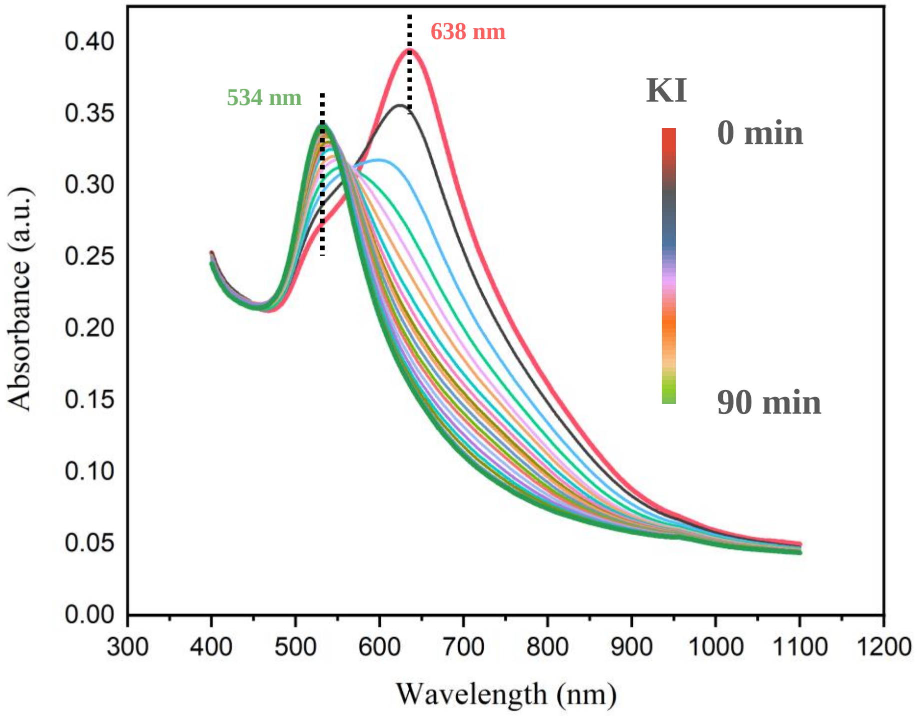

Figure S1. The UV-Vis spectra of GNS solution in the presence of 2 μM KI after various periods (0-90 min) of reaction time.

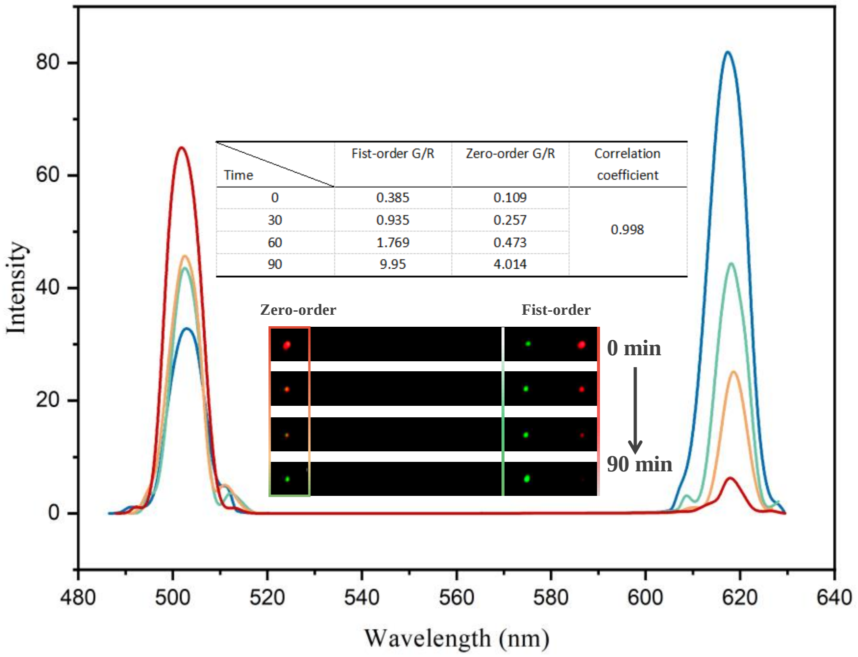

Figure S2. Representative G/R ratios and scattering spectra of single GNSs irradiated with R and G lasers. Exposure time: 50 ms; reaction time: 0, 30, 60, and 90 min. Inset: Corresponding DFM images of single GNSs after treatment with 0.5 μM KI, showing a color change from red to green.

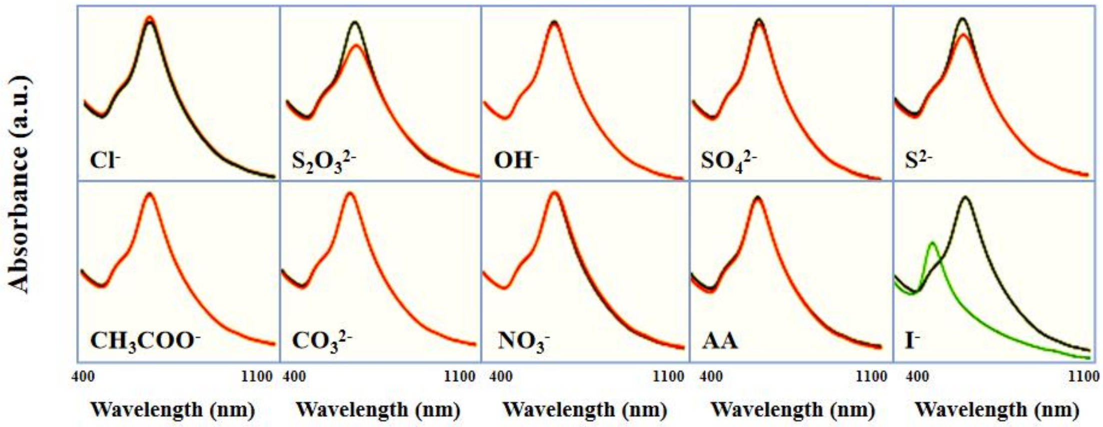

Figure S3. UV-Vis spectra of the selective response of GNSs with the presence of different anions, including Cl^-, $S_2O_3^{2-}$, OH^-, SO_4^{2-}, S^{2-}, CH_3COO^-, CO_3^{2-}, NO^{3-} and AA.

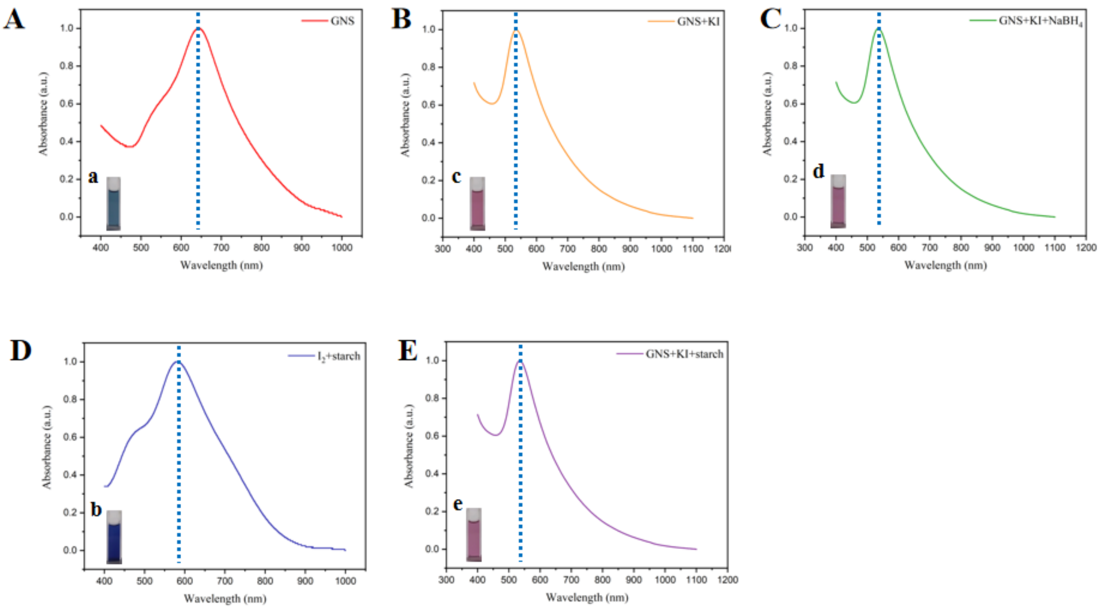

Figure S4. Photographs and UV-vis spectra of GNSs (**A**), and in the presence of 2 μM KI (**B**), 2 μM KI and 100 μM $NaBH_4$ (**C**),100 μM I_2 that had reacted with 0.5% starch for 3 min (**D**), and 2 μM KI and 0.5% starch.

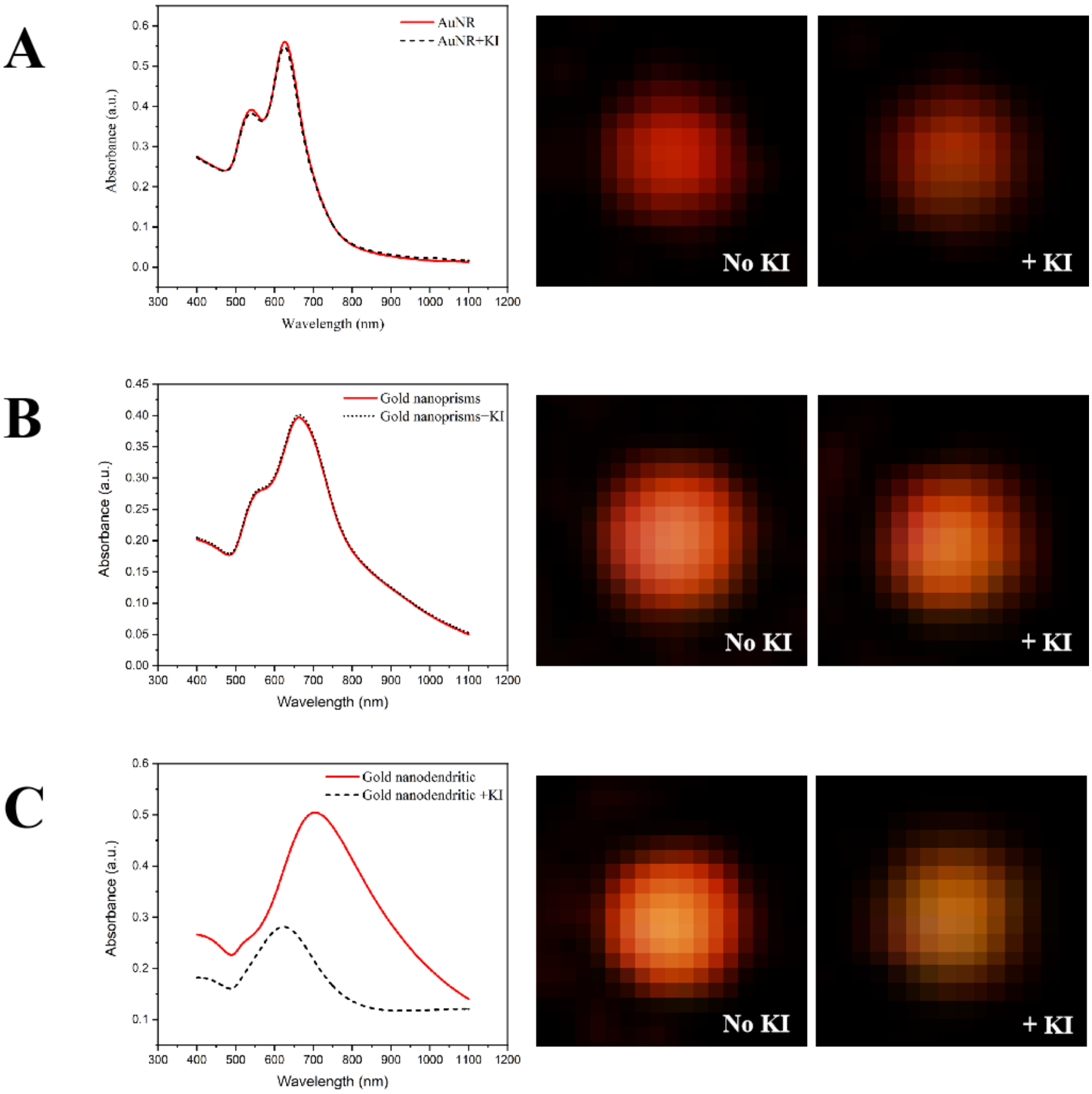

Figure S5. UV-Vis spectra and DFM images of Au nanorods (A), Au nanoprisms (B), and larger GNSs capped with citrate (C) in the presence of 2 μM KI.
